# Impact of Vietnam-era G.I. Bill eligibility on the distribution of later-life memory score levels and decline: evidence from the Vietnam draft lottery natural experiment

**DOI:** 10.1093/aje/kwag097

**Published:** 2026-05-08

**Authors:** Aayush Khadka, Lucia Pacca, Jillian Hebert, Justin S White, Rita Hamad, Willa D Brenowitz, M Maria Glymour, Anusha M Vable

**Affiliations:** Department of Family and Community Medicine, University of California San Francisco, San Francisco, CA, United States; Philip R. Lee Institute for Health Policy Studies, University of California San Francisco, San Francisco, CA, United States; Department of Family and Community Medicine, University of California San Francisco, San Francisco, CA, United States; Philip R. Lee Institute for Health Policy Studies, University of California San Francisco, San Francisco, CA, United States; Department of Epidemiology and Biostatistics, University of California San Francisco, San Francisco, CA, United States; Department of Family and Community Medicine, University of California San Francisco, San Francisco, CA, United States; School of Public Health, Washington University in St. Louis, St. Louis, MO, United States; Department of Health Law, Policy and Management, Boston University, Boston, MA, United States; Department of Social and Behavioral Sciences, Harvard T. H. Chan School of Public Health, Boston, MA, United States; Department of Epidemiology and Biostatistics, University of California San Francisco, San Francisco, CA, United States; Kaiser Permanente Center for Health Research, Portland, OR, United States; Department of Epidemiology, Boston University, Boston, MA, United States; Department of Family and Community Medicine, University of California San Francisco, San Francisco, CA, United States; Philip R. Lee Institute for Health Policy Studies, University of California San Francisco, San Francisco, CA, United States; School of Public Health, Washington University in St. Louis, St. Louis, MO, United States

**Keywords:** cognitive aging, distributional effects, quantile regressions, natural experiment, Vietnam G.I. Bill, Vietnam draft lottery

## Abstract

We estimated the distributional impact of Vietnam-era G.I. Bill eligibility, a federal policy that subsidized education, impacting approximately 6 million veterans, on later-life memory levels and rate of age-related decline. We used Health and Retirement Study data (1998–2018) for men born 1947–1953 with at least 1 outcome assessment (*N* = 2337; 15 433 person-waves). We proxied Bill eligibility using draft eligibility based on birthdate and self-reported veteran status. We used an algorithmic measure of memory as the outcome. We fit intention-to-treat and instrumental variables models at the mean and different outcome quantiles. We assessed effect modification using a childhood socioeconomic status index and self-reported race and ethnicity. Although imprecise, results are consistent with eligibility increasing memory levels but accelerating age-related decline, especially at lower quantiles. The magnitude of estimated effects was especially pronounced for men from low childhood socioeconomic backgrounds and Black men. For White men, eligibility was associated with uniformly accelerated decline across the distribution. Overall, our results paint a complex picture: eligibility may have improved overall memory score levels but also may have slightly increased the rate of age-related decline, especially among those with worse memory function. Further research is necessary to investigate the mechanisms by which such policies impact later-life memory.

## Introduction

Alzheimer’s disease-related dementias (ADRD) is a major public health challenge in the United States: some estimates suggest that more than 7.2 million people are currently impacted by ADRD and that by 2060, 14 million US residents aged 65 and older will be living with the disease.^1^^,^^2^ Population level solutions, especially ones that can shift and reshape the distribution of risk factors as conceptualized by Rose and Razak et al., have the potential to mitigate ADRD risk.^3^^,^^4^ Particularly promising population level solutions for ADRD may be policies that improve education levels as increased educational attainment has been shown to improve cognitive function in old age.

In this study, we exploit a natural experiment due to the Vietnam draft lottery to identify the impact of the Vietnam-era G.I. Bill on later-life memory, a key component of cognitive function. The Vietnam-era G.I. Bill was a federal policy that was signed into law in 1966 and impacted around 6 million veterans up until the 1980s, primarily by subsidizing their college education.^5^^-^^8^ To assess the impact of the policy, we focus on being eligible for the G.I. Bill benefits as opposed to actually receiving the Bill’s benefits. Eligibility for G.I. Bill benefits depended on the dates, duration, and discharge method from military service.^9^^,^^10^ Approximately, 72% of eligible veterans had used the Bill’s benefits by 1984.^11^ While the G.I. Bill increased college attendance, access to these benefits varied widely by race and socioeconomic status.^8^^,^^12^^,^^13^

G.I. Bill eligibility and benefits have been linked to improvements in risk factors for later-life cognitive function such as education levels and blood pressure^12^^,^^14^; however, whether these improvements will translate to improvements in both memory levels and rate of age-related decline is unclear. For example, higher education has primarily been linked to increased average cognitive levels and not necessarily with any changes to the rate of age-related cognitive decline.^15^^-^^17^ At the same time, a new line of inquiry is showing that increased college attendance—as accomplished by the G.I. Bill—without subsequently increasing college graduation rates may not translate to health improvements in later life.^12^^,^^18^ Finally, since eligibility for and access to the Bill’s benefits varied by race, ethnicity, and socioeconomic status,^14^ the effects of the G.I. Bill eligibility differs across individual backgrounds.

## Methods

### Data

We used restricted access data from the Health and Retirement Study (HRS), a nationally representative, biennial, longitudinal survey of noninstitutionalized US adults aged 50+ and their spouses (study period: 1998–2018). The HRS collects information on respondent demographics, physical health, cognitive health, and economic status. It also collects responses via proxy (typically spouses or caregivers) for those who are unwilling or unable to answer. The restricted access data includes each respondent’s exact date of birth.

We also used data on the Vietnam draft lottery numbers, that is, the ranking of each date of birth for the draft. These numbers are publicly available for download from the US Selective Service System website.^19^ We received permission from the HRS administrators to link the draft lottery data to the restricted HRS data using a respondent’s exact date of birth.

### Analytic sample

We merged HRS data with draft lottery numbers using each respondent’s exact date of birth. All men born between 1944 and 1956 were eligible for inclusion in our study as they were affected by the lottery (*N* = 4212). We restricted the eligible sample to men born between 1947 and 1953 because the lottery most strongly predicted military service for these men (Table S1; 1828 men dropped, *N* = 2384 remaining). We additionally restricted the data to individuals who had at least 1 outcome measure over the study period (47 men dropped). Upon applying these inclusion criteria, our analytic sample had 2337 unique respondents contributing 15 433 person-waves of data (median follow-up time: 4 waves or approximately 8 years).

### Vietnam draft lottery

Between 1969 and 1975, to increase troop size and ensure some degree of fairness in recruitment, the US Selective Service System held annual draft lotteries applicable only to men born in certain birth cohorts.^20^ In each lottery, eligible men were assigned a random number between 1 and 366 based on their date of birth (Table S2). The military would then select a threshold number based on its needs and all men with lottery numbers less than or equal to the threshold were deemed draft-eligible (Table S2 also lists the threshold numbers).

### Exposure

We were unable to directly identify G.I. Bill-eligible individuals in the HRS due to lack of information on manner of discharge from the military. Therefore, we proxied G.I. Bill eligibility in 2 ways: first, as an indicator variable, draft eligibility, that took the value 1 if an individual’s lottery number, based on his birthdate, was less than the threshold value for the relevant lottery, and 0 otherwise. The threshold value varied by year and was determined based on how many men the military needed. Second, as self-reported military service, an indicator variable that equaled 1 if a respondent reported having served in the military at any time during their life, and 0 otherwise.

### Outcome

Our primary outcome was an algorithmically defined memory score developed by Wu and colleagues (2013) in the HRS.^21^ Memory scores were predicted using a linear regression model and accounted for each respondent’s responses to cognitive questions as well as various demographic characteristics. The regression model had an R-squared value of 0.61 among participants 70+ years old.

Memory score was available for both direct and proxy respondents, which allowed us to include the most cognitively impaired individuals (ie, those who were unable to do the interviews themselves and had to rely on a proxy) in our analysis. Lower values represented worse memory. For the analysis, we standardized the outcome to the 1998 memory score distribution in our sample to aid with interpreting regression coefficients.

### Covariates

In all regressions, we controlled for practice effects to account for improved performance on cognitive tests due to increased familiarity with the testing instrument over time in longitudinal studies.^22^ We defined practice effects as an indicator variable that took the value 1 the first time someone was responding to cognitive questions, and 0 otherwise.^23^ We also controlled for month-and-year-of-birth fixed effects, that is, an indicator variable for each unique month-and-year-of-birth combination in our data (eg, January 1947, February 1947). These fixed effects allowed us to control for potential secular trends in the outcome while also accounting for a mechanical artifact with the randomization in the 1969 lottery, which led to lower lottery numbers being assigned with higher probability to those born later in the year.^24^^,^^25^

### Effect modifier

We studied effect modification by childhood socioeconomic status (cSES) and race and ethnicity. We used a previously validated index developed in the HRS to measure cSES.^26^ This index accounts for measures of childhood human, financial, and social capital.^27^ We created tertiles of the cSES index representing low, medium, and high cSES. Those with missing cSES index values were dropped from the primary effect modification analysis. Race and ethnicity were self-reported by HRS respondents (non-Hispanic White, non-Hispanic Black, non-Hispanic Other races, Hispanic). We did not estimate effect modification in the “Other races” group as this constituted 3.6% of the analytic sample and because interpreting results for such a heterogeneous group is difficult.

### Measure of time

To model memory score decline, we used the number of survey waves (approximating a 2-year interval) since baseline as our measure of time. Baseline was defined as either the start of the study period (1998 wave) or the first time a respondent completed an HRS survey. We controlled for baseline age (centered at 60 years) in our regression models.

### Missing data

We assessed the proportion of missing observations for variables used in our analysis, stratified by draft eligibility. Since item missingness was nondifferential by draft eligibility status (Table S3), we did not impute missing data.

### Loss to follow-up

We assessed the proportion of respondents lost to follow-up (death or dropped from the survey for other reasons) by draft eligibility. Loss to follow-up was also nondifferential by draft eligibility (Table S4); thus, we did not address it in our statistical analysis.

### Statistical analysis

#### Summary statistics

To describe their distribution and assess covariate balance by draft eligibility and self-reported military service, we estimated summary statistics of race and ethnicity, year of birth, maternal and paternal education (0–17+, where 17+ represents 17 or more years of schooling), birth in the US South (indicator variable), birth in the United States (indicator variable), and cSES index. We also estimated average follow-up time by draft eligibility status.

#### Intention-to-treat effect

To estimate draft eligibility’s impact on memory score levels at the mean and at each unit quantile from the 1st–99th quantiles of the outcome distribution, we fit the following models:


(1) \begin{equation*} E\left[{Y}_{it}|{Z}_i,{W}_{it},{P}_{it},{X}_i,{\delta}_i\right]={\beta}_0+{\beta}_1{Z}_i+{\pi W}_{it}+\lambda{P}_{it}+\gamma{X}_i+{\delta}_i \end{equation*}

(2) \begin{equation*} {Q}_{\tau}\left({Y}_{it}|{Z}_i,{W}_{it},{P}_{it},{X}_i,{\delta}_i\right)={\alpha}_{0,\tau }+{\alpha}_{1,\tau }{Z}_i+{\pi}_{\tau }{W}_{it}+{\lambda}_{\tau }{P}_{it}+{\gamma}_{\tau }{X}_i+{\delta}_i \end{equation*}

In both equations, the subscripts *i* and *t* represent the respondent and time, ${Y}_{it}$ represents standardized memory score, ${Z}_i$ represents an individual’s draft eligibility status, ${W}_{it}$ represents the number of waves since baseline, ${P}_{it}$ represents practice effects, ${X}_i$ represents baseline age, and ${\delta}_i$ represents month-and-year-of-birth fixed effects. Eqn (2) additionally indexes each coefficient with *τ*, which indicates the quantile at which the equation is being estimated. In eqn (1), ${\beta}_1$ represents draft eligibility’s impact on average standardized memory scores over the entire study period. Similarly, in eqn (2), ${\alpha}_{1,\tau }$ represents draft eligibility’s impact on the ${\tau}^{th}$ quantile of the standardized memory score distribution over the entire study period.

We estimated the intention-to-treat effect on memory score decline using the following equations:


(3) \begin{equation*} E\left[{Y}_{it}|{Z}_i,{W}_{it},{P}_{it},{X}_i,{\delta}_i\right]={\beta}_0+{\beta}_1{Z}_i+{\beta}_2{Z}_i{W}_{it}+{\pi W}_{it}+\lambda{P}_{it}+\gamma{X}_i+{\delta}_i \end{equation*}

(4) \begin{equation*} {Q}_{\tau}\left({Y}_{it}|{Z}_i,{W}_{it},{P}_{it},{X}_i,{\delta}_i\right)={\alpha}_{0,\tau }+{\alpha}_{1,\tau }{Z}_i+{\alpha}_{2,\tau }{Z}_i{W}_{it}+{\pi}_{\tau }{W}_{it}+{\lambda}_{\tau }{P}_{it}+{\gamma}_{\tau }{X}_i+{\delta}_i\kern0.5em \end{equation*}

These two equations resemble eqns (1) and (2) in almost all ways except for the inclusion of the interaction term between ${Z}_i$, the draft eligibility indicator, and ${W}_{it}$, the number of waves since baseline. In eqn (3), ${\beta}_2$ represents the additional change in the average rate of age-related memory score decline due to draft eligibility. Similarly, in eqn (4), ${\alpha}_{2,\tau }$ represents the additional change in the rate of age-related memory score decline at the ${\tau}^{th}$ quantile of the memory score distribution due to being draft eligible.

We estimated eqns (1) and (3) using Generalized Estimating Equations with an independent correlation structure and robust standard errors.^28^^,^^29^ We estimated eqns (2) and (4) using the Machado and Santos Silva quantile regression estimator.^30^ We illustrated the distributional effect of draft eligibility on memory score over time using our estimated intention-to-treat effects from eqn (4).^31^

#### Instrumental variables analysis

The local average treatment effect (LATE) represents the impact of military service on memory score levels and decline among compilers, that is, men who served because they were deemed draft-eligible by the lottery but would not have done so otherwise. To estimate the LATE, we constructed 2 instruments: (1) the draft eligibility indicator variable and (2) an interaction of the draft eligibility indicator variable with the number of waves in the study since baseline.

We tested the strength of these instruments in predicting military service and the interaction of military service with time in the study to test for the first-stage relationship. We assumed that the instruments impacted memory score only through its effect on military service, which may be reasonable since draft avoidance during the lottery period was relatively low.^12^ We additionally assumed that the instruments were independent of potential outcomes conditional on month-and-year-of-birth fixed effects, which is reasonable given that the instrument is a randomization indicator. Finally, we assumed that draft eligibility did not decrease the probability of anyone serving in the military.

We used the 2-stage least squares (2SLS) estimator to estimate the LATE using the following second-stage equations:


(5) \begin{equation*} E\left[{Y}_{it}|\overset{\sim }{M_i},{W}_{it},{P}_{it},{X}_i,{\delta}_i\right]={\beta}_0+{\beta}_1{\overset{\sim }{M}}_i+{\pi W}_{it}+\lambda{P}_{it}+\gamma{X}_i+{\delta}_i\kern1em \end{equation*}

(6) \begin{equation*} E\left[{Y}_{it}|\overset{\sim }{M_i},{W}_{it},{P}_{it},{X}_i,{\delta}_i\right]={\beta}_0+{\beta}_1{\overset{\sim }{M}}_i+{\beta}_2\overset{\sim }{M_i{W}_{it}}+{\pi W}_{it}+\lambda{P}_{it}+\gamma{X}_i+{\delta}_i\kern0.5em \end{equation*}

In eqns (5) and (6), ${\overset{\sim }{M}}_i$ represents military service that has been instrumented using the draft eligibility indicator variable. In eqn (6), $\overset{\sim }{M_i{W}_{it}}$ represents the interaction of military service and number of waves in the study since baseline, instrumented by the interaction between draft eligibility and number of waves in the study since baseline. All other covariates are the same as in the previous equations. In eqn 5, ${\beta}_1$ is our coefficient of interest whereas in eqn (6), ${\beta}_2$ is our coefficient of interest. For statistical inference, we estimated the Huber-White cluster-robust SEs clustered at the individual level in both equations. We evaluated the strength of the instrumental variable using the partial F-statistic. Additionally, we conducted a nonparametric test of the exclusion restriction and monotonicity of an instrumental variable proposed by Frandsen et al.^32^

Using the same 2 instruments, we estimated quantile treatment effects (QTE) of military service on memory scores and decline at the median of the memory score distribution. We used the Kaplan and Sun smoothed estimating equations method to fit the following instrumental variables quantile regression models.^33^ We estimated QTEs only at the median because they serve as a useful comparator for mean effects and because we experienced several model convergence issues at other quantiles. We estimate the QTEs using the following equations:


(7) \begin{equation*} {Q}_{\tau}\left({Y}_{it}|{\overset{\sim }{M}}_i,{W}_{it},{P}_{it},{X}_i,{\delta}_i\right)={\alpha}_{0,\tau }+{\alpha}_{1,\tau }{\overset{\sim }{M}}_i+{\pi}_{\tau }{W}_{it}+{\lambda}_{\tau }{P}_{it}+{\gamma}_{\tau }{X}_i+{\delta}_{i,\tau}\kern1.5em \end{equation*}

(8) \begin{align*} &{Q}_{\tau}\left({Y}_{it}|{\overset{\sim }{M}}_i,{W}_{it},{P}_{it},{X}_i,{\delta}_i\right)={\alpha}_{0,\tau }+{\alpha}_{1,\tau }{\overset{\sim }{M}}_i+{\alpha}_{2,\tau }\ \overset{\sim }{M_i{W}_{it}}+{\pi}_{\tau }{W}_{it}+{\lambda}_{\tau }{P}_{it}\nonumber\\&+{\gamma}_{\tau }{X}_i+{\delta}_{i,\tau}\kern0.75em \end{align*}

The variables included in eqns (7) and (8) are defined the same way as variables included in eqns (5) and (6). *τ* indexes the quantile of interest and equals 0.5 in both equations to represent the median. In eqn (7), ${\alpha}_{1,\tau }$ is our coefficient of interest, whereas in eqn (8), ${\alpha}_{2,\tau }$ is our coefficient of interest. The estimated treatment effects from both equations apply to the entire population represented by our sample, that is, they are not LATE estimates.

#### Effect modification

For our effect modification analysis, we stratified our analytic sample by levels of the effect modifier and estimated eqns (1)–(8) in each stratum of the effect modifier.

#### Sensitivity analyses

We conducted 3 sensitivity analyses. First, to assess if our analytic sample inclusion criteria affected our results, we fit eqns (1)–(8) in a sample of all men born between 1944 and 1953 who had the outcome measured at least once. Second, to assess the robustness of our use of self-reported veteran status as a proxy, we redefined military service as an indicator variable that equaled 1 only if a respondent served during the lottery years and 0 otherwise. Finally, to assess the robustness of our conclusions to the outcome definition, we redefined the outcome as total word recall score, which we defined as the sum of immediate and delayed word recall scores. Since immediate and delayed word recall were cognitive tests administered only to direct HRS respondents, we did not have data on total word recall score for proxy respondents.

### Statistical software

We used Stata/MP 18.0 for all analyses.^34^ We fit the Machado and Santos Silva quantile regression estimator using the “xtreg” package and the instrumental variables quantile regression model using the “ivqregress” package with the “smooth” estimator.^35^^,^^36^ We created all figures in RStudio.^37^

### Data and code availability

Using restricted access HRS data requires permission from the HRS administrators. Vietnam draft lottery numbers are publicly available from the US Selective Service System website.^19^ All statistical code was reviewed by the second author following recommended practice.^38^ In compliance with the data usage agreement with the Health and Retirement Study, data cannot be made publicly available; redacted code will be made available on the senior author’s lab’s GitHub page.

### Results

#### Summary statistics

Around 42% of our analytic sample were draft-eligible (Table 1). Draft-eligible and ineligible groups were similar in terms of most demographic characteristics including average memory score over the study period. Relative to nonveterans, a greater proportion of veterans reported being non-Hispanic White, being born in the United States, being born in a Southern state, and being born between 1947 and 1950. Average memory score was roughly the same between veterans and nonveterans.

**Table 1:** Distribution of covariates in the primary analytic sample overall and disaggregated by draft eligibility and self-reported military service.

		**Draft-eligible**		**Self-reported military service**	
	**Overall (*N* = 2337)**	**Yes (*N* = 983)**	**No (*N* = 1354)**	**Veteran (*N* = 745)**	**Nonveteran (*N* = 1592)**
Veteran (%)	31.9	40.4	25.7		
Race and ethnicity (%)					
Non-Hispanic White	58.7	60.6	57.3	64.3	56.0
Non-Hispanic Black	21	20.5	21.4	24.0	19.6
Non-Hispanic Other	3.6	2.7	4.3	2.4	4.2
Hispanic	16.7	16.3	16.9	9.3	20.2
Maternal education (years)	10.3	10.3	10.3	10.9	10.0
Paternal education (years)	10	10.2	9.9	10.3	9.9
Southern birth (%)	31.4	29.9	32.5	36.6	29.0
US birth (%)	84.7	85.6	84.1	96.8	79.1
Birth year (%)					
1947	10.4	13.1	8.3	16.5	7.5
1948	13.1	16	11.1	17.9	10.9
1949	14.3	18.4	11.3	19.1	12.1
1950	15.2	19.6	12	15.7	14.9
1951	14	12.4	15.1	10.1	15.8
1952	16.5	10.4	21	11.0	19.1
1953	16.5	10.1	21.2	9.8	19.7
Baseline age (years)	54.9	55.2	54.6	55.1	54.7
Childhood socioeconomic status index	0.3	0.3	0.3	0.3	0.3
Average time in study (waves)	3.8	3.8	3.8	3.8	3.8
Average memory score	−0.4	−0.4	−0.3	−0.4	−0.3

Draft eligibility is defined based on respondent’s exact date of birth and lottery number assigned to them by the Vietnam draft lottery. The primary analytic sample consists of all men in the Health and Retirement Study born between 1947 and 1953 and who met the other inclusion criteria for our study.

#### Intention-to-treat effects

Compared to draft-*ineligible* veterans, draft-eligible veterans had higher memory score levels by 0.02 SD (95% CI, –0.04 to 0.08) at the mean (Figure 1A), 0.03 SD (95% CI, –0.04 to 0.10] at the 10th quantile, and 0.01 SD (95% CI, –0.02 to 0.04) at the median, but lower levels at the 90th quantile (–0.01 SD [95% CI, –0.06 to 0.04]).

**Figure 1:**
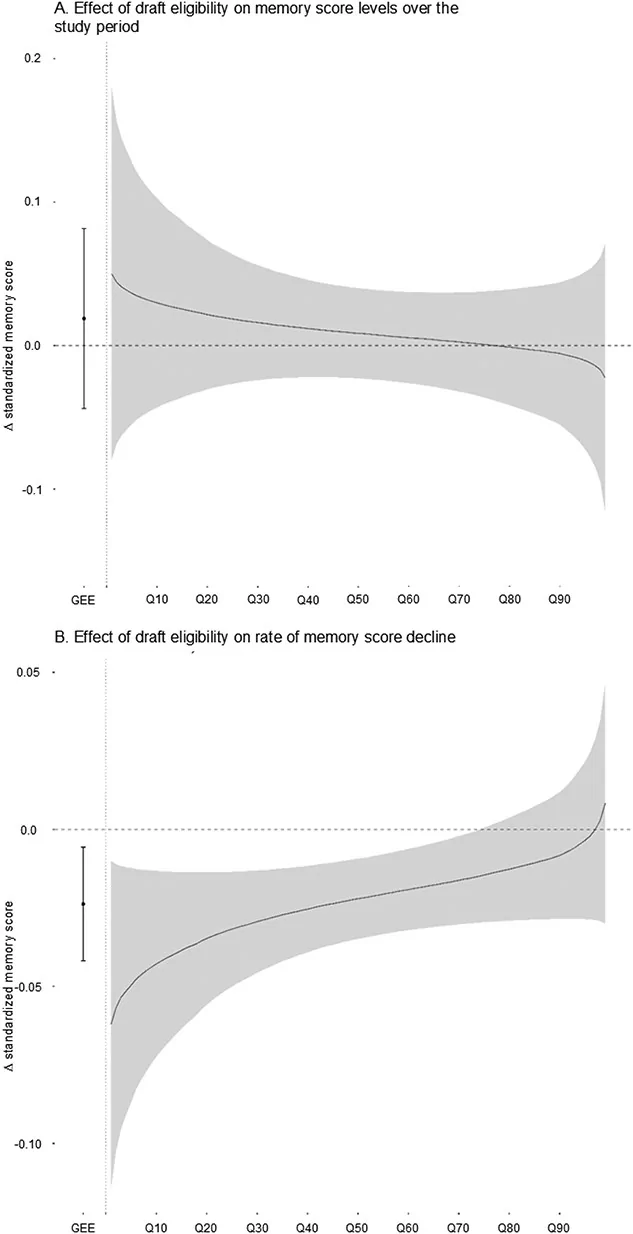
Intention-to-treat effect on memory score levels and rate of memory score decline at the mean and at quantiles of interest. Notes: Both panels illustrate the intention-to-treat effects at the mean (“GEE”) and between the 1st and 99th quantiles (quantiles on the *x*-axis labeled as “Q10” for the 10th quantile, “Q20” for the 20th quantile, and so forth). The *y*-axis for both panels represents the change in standardized memory score, that is, the coefficient from regression models. (A) shows draft eligibility’s effect on memory score levels and (B) shows draft eligibility’s effect on the rate of memory score decline. Effects at the mean were estimated using generalized estimating equations while quantile regression estimates were produced using the Machado and Santos Silva quantile regression estimator. All models controlled for practice effects, year in which the outcome was measured, and month-and-year-of-birth fixed effects.

In contrast, for each additional wave in the study, draft eligibility was associated with faster memory score decline by –0.02 SD per wave (95% CI, –0.04 to –0.01) at the mean (Figure 1B), –0.04sd per wave (95% CI, –0.07 to –0.01) at the 10th quantile, –0.02 SD per wave (95% CI, –0.03 to –0.01) at the 50th quantile, and –0.01 SD per wave (95% CI, –0.03 to –0.01) at the 90th quantile. Thus, over time, draft-eligible veterans ended up having lower memory score levels compared to draft-ineligible veterans (Figure 2).

**Figure 2:**
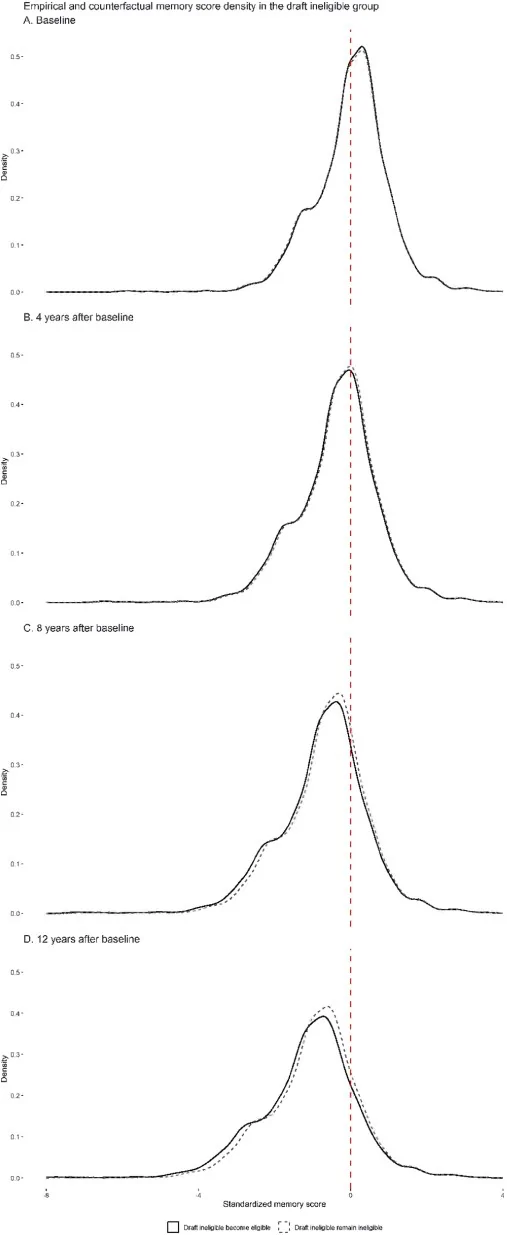
Implied reshaping of the memory score distribution by our intention-to-treat effects estimated from our intention-to-treat model of memory score decline. Notes: The solid black density plot shows the memory score distribution in the draft ineligible group if it remains draft-ineligible. The dashed gray density plot shows the memory score distribution the draft-ineligible group would have had if it had been draft-eligible from the beginning of our study period. (A) presents empirical distribution of memory score in the draft ineligible group in solid black and the implied change in memory distribution if the draft ineligible group became eligible in dashed gray. (B–D) illustrates the changes in these 2 distributions 4, 8, and 12 years after baseline. The dashed red vertical line going across the plots orients readers to the 0 value for standardized memory score. At baseline, the draft-ineligible group would have had slightly higher memory scores if it had been draft-eligible, as indicated by the solid black density plot being slightly to the right of the dashed gray density plot. Over time, both distributions move to the left, indicating declining memory scores with age; however, being draft-eligible accelerates the decline, which is shown by the solid black density plot starting to move toward the left of the dashed gray density plot over time.

#### Instrumental variables effects

Draft eligibility was a reasonably strong instrument with a first-stage partial F-statistic of 18.89 (Table 2). The nonparametric test for the exclusion restriction and monotonicity suggested that the instrument satisfied both assumptions (*P* = 1.00). Among men who served in the military because of being draft-eligible, but who would not have otherwise served, military service was estimated to increase average memory score levels (from eqn (5), ${\beta}_1$ = 0.11 SD [95% CI, –0.52 to 0.73]), although CIs were very wide. Military service also accelerated the rate of average memory score decline (from eqn (6), ${\beta}_2$ = –0.14 SD per wave [95% CI, –0.26 to 0.03]) (Table 2). Military service also increased median memory score levels (from eqn (7), ${\alpha}_{1,\tau }$ = 0.13 SD [95% CI, –0.09 to 0.34]) and accelerated the rate of median memory score decline (from eqn (8), ${\alpha}_{2,\tau }$ = 0.09 SD per wave [95% CI, –0.16 to –0.02]) (Table 2).

**Table 2:** Impact of instrumented veteran status on standardized memory score levels and rate of decline.

	**Point estimate**	**95% CI**
Local average treatment effect		
Memory score levels	0.11	[–0.52 to 0.73]
First-stage partial F-statistic: 18.89		
Rate of memory score decline	–0.14	[–0.26 to –0.03]
Impact on median memory score		
Memory score levels	0.13	[–0.09 to 0.34]
Rate of memory score decline	–0.09	[–0.16 to –0.02]
Total person-wave observations	13 031	

Instrumental variables analysis results are presented both for mean memory score (ie, the local average treatment effect) and median memory score. The impact at the median is not applicable to just the complier population but to the entire sample.

#### Effect modification analysis

- i) Childhood socioeconomic status

### Intention-to-treat effects

Draft eligibility increased average memory score levels in all 3 cSES groups, with the largest increases in low-cSES men. Draft eligibility also increased memory score levels the most at lower quantiles for low- and high-cSES men; however, among men with medium cSES backgrounds, draft eligibility decreased memory score levels at lower quantiles (Figures S1–S3).

Draft eligibility also accelerated the average rate of memory score decline by approximately the same amount among low- and medium-cSES men, but less so among high-cSES men (Figures S4–S6). Furthermore, among low- and medium-cSES men, draft eligibility increased the rate of memory score decline at lower memory quantiles; however, among high-cSES men, draft eligibility increased the rate of memory score decline more at higher memory quantiles.

### Instrumental variable effects

Military service impacted average and median memory score levels and decline in the same direction as draft eligibility effects in each stratum of cSES, although the point estimates were larger and the confidence intervals were substantially wider (Table S5).

- ii) Race and ethnicity

### Intention-to-treat effect

Draft eligibility increased average memory score levels among Black and Hispanic men, but not among White men (Figures S7–S9), although confidence intervals were wide. Effects across the memory score quantiles were in the same direction and uniform for Black and Hispanic men; however, among White men, draft eligibility increased memory score levels at lower quantiles, but decreased them at higher quantiles (Figures S7–S9).

Among White men, draft eligibility accelerated the rate of memory score decline roughly uniformly across the entire outcome distribution (Figure S10). For Black (Figure S11) and Hispanic men (Figure S12), draft eligibility increased the rate of memory score decline at lower quantiles and at the mean but slowed the rate of decline at higher quantiles.

### Instrumental variable effects

For White men, military service appeared not to impact average memory score levels, but increased average rate of memory score decline, median memory score levels, and the rate of decline in memory score at the median (Table S6). In Black and Hispanic men, results from the instrumental variables analysis had very wide CIs (Table S6).

#### Sensitivity analyses

In the sample of men born between 1944 and 1953, intention-to-treat effects and instrumental variable effects were mostly similar to results from our primary analysis (Table S7; Figure S13). One difference was that intention-to-treat effects on memory score decline was approximately the same across the entire outcome distribution.

When we additionally constrained the veteran status variable to include only those who started serving during the lottery years, we found that the instrumental variables results were consistent with the primary analysis in terms of both magnitude and direction (Table S8).

When using total word recall score as the outcome, intention-to-treat effects at the mean were similar to the primary analysis (Table S9; Figure S14). Draft eligibility increased total word recall scores at lower outcome quantiles but decreased total word recall scores at higher outcome quantiles; however, draft eligibility’s effect on the rate of total word score decline was approximately the same across the entire outcome distribution. Instrumental variable effects at the mean and median were in the same direction as the intention-to-treat effects.

## Discussion

In this study, we exploited a natural experiment due to the Vietnam draft lottery to examine the long-run, cognitive consequences of Vietnam-era G.I. Bill eligibility. While our estimates were generally imprecisely estimated, the direction of the point estimates were consistent with the following: G.I. Bill eligibility was associated with modestly higher memory score levels but also slightly accelerated memory score decline, especially at the lower memory score quantiles. Taken together, these results indicate that G.I. Bill eligibility shifted the level of memory score upward, but over time, these gains in memory score levels were diminished. We also found that G.I. Bill eligibility had the strongest effects for men from low-cSES backgrounds. Finally, we found that for Black and Hispanic men, G.I. Bill eligibility appeared to accelerate memory score decline the most at lower quantiles but for White men, the acceleration in memory score decline appeared to be uniform across the outcome distribution.

Our main result—that G.I. Bill eligible veterans had higher memory score levels but potentially faster rates of decline—is generally consistent with results from the education-cognitive aging literature, suggesting G.I. Bill eligibility may have impacted later-life memory primarily through increased college education.^15^^-^^17^ Interestingly, we found that these effects were stronger at lower memory score quantiles relative to higher quantiles. However, such interpretations of our results rest on our assessment of the validity of draft eligibility and veteran status as proxies for G.I. Bill eligibility. Military service may be a good proxy for G.I. Bill eligibility as it was a prerequisite for becoming eligible, and draft eligibility may also be a good proxy as it strongly predicted military service, especially in our data. Furthermore, the lottery occurred at a period where deferments via college enrollments were minimal, meaning that our reduced form estimates should not be impacted by differential attrition. However, G.I. Bill eligibility also depended on the dates, duration, and discharge status of military service, which limits the reliability of military service as a proxy for G.I. Bill eligibility.

Additionally, our analysis cannot conclusively establish if there were pathways other than through college education that really impacted our results. As shown in Figure 3, military service could have directly impacted later-life cognition through, for example, participation in active duty service, exposure to environmental toxins, or intense psychological stressors associated with service in the Vietnam era, and the challenges veterans faced upon returning home. Similarly, military service also gives veterans access to healthcare benefits through the Veterans Administration, which could directly impact later-life cognitive function and other health outcomes. It is likely that our primary effect estimates reflect a mix of all these pathways. Furthermore, our effect modification results may suggest that different pathways may be differentially important for men from marginalized socioeconomic backgrounds and for individuals with worse vs better memory. For example, the larger effects on memory score levels and decline for men from low-cSES backgrounds may reflect that for these individuals, the impact of the proxies operated primarily from the college education pathway.

**Figure 3:**
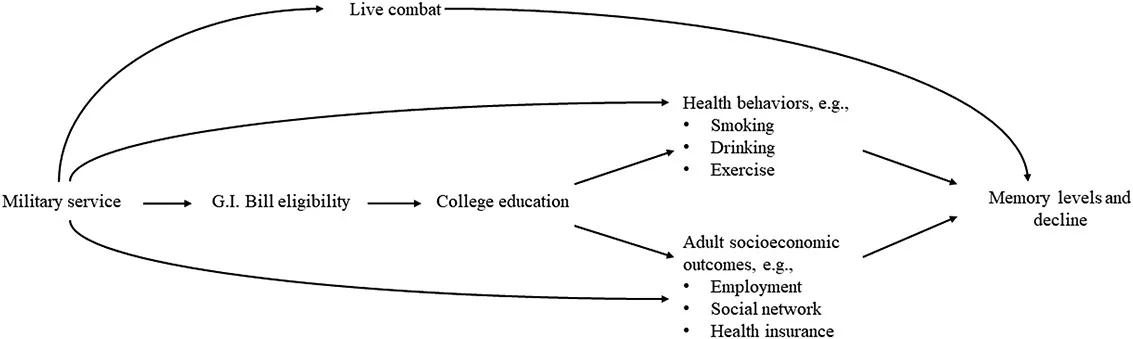
Mechanisms linking Vietnam-era G.I. Bill eligibility with later-life memory score levels and rate of decline.

These findings need to be viewed in light of our study’s limitations. First, the memory score outcome values were predicted using an algorithm applied to the HRS data. The advantage of this algorithm is that it used immediate and delayed word recall scores and predicted memory scores for those who responded via proxy. However, the algorithm also has key drawbacks: first, the model was trained on data from 70+-year-olds only; second, the predictive power of the model was somewhat limited (*R*-squared = 0.61); and third, it is not clear if the model includes all relevant covariates for capturing the underlying construct of memory. Second, our results may lack generalizability because our sample included men born in specific birth cohorts. Therefore, our results may not apply to women, who evidence suggests benefit more from educational attainment than men, or to men from earlier or later birth cohorts. Third, we modeled memory score decline linearly for simplicity; however, this modeling choice may not capture the true relationship between education and memory score decline in later life. Finally, despite the inclusion of proxy respondents, we cannot rule out the possibility of differential attrition by levels of memory score, which may induce attrition bias into our estimates.

## Conclusions

The pattern of point estimates from our analysis suggests that Vietnam-era G.I. Bill eligibility may have modestly increased later-life memory score levels while concurrently accelerating age-related memory score decline. Although substantial uncertainty around these estimates limits our ability to come to strong conclusions, the consistency of the direction of the estimates across analytic approaches and subgroups provides evidence of this trade-off between higher memory levels and faster decline. Future work should seek to replicate these findings in larger samples and more directly disentangle the pathways by which G.I. Bill eligibility may have shaped later-life memory through mediation analyses or structural equation modeling.

## Supplementary Material

Web_Material_kwag097

## Data Availability

Restricted Health and Retirement Study data can be accessed upon receiving approval from the study’s administrators. Vietnam draft lottery number data can be downloaded from the US Selective Service System website. In compliance with the data usage agreement with the Health and Retirement Study, redacted code will be made available on the senior author’s lab’s GitHub page.
