# Supplementary material for: Impact of Vietnam-era G.I. Bill eligibility on the distribution of later-life memory score levels and decline: evidence from the Vietnam draft lottery natural experiment
Source: Am J Epidemiol. 2026 May 8;195(11):2880–8. doi: 10.1093/aje/kwag097 (PMC13361569; doi:10.1093/aje/kwag097)
Supplement: Supplementary file 1 [file web_material_kwag097.docx]

**Manuscript title**	Impact of Vietnam-era G.I. Bill eligibility on the distribution of later-life memory score levels and decline: Evidence from the Vietnam draft lottery natural experiment

**Authors**	Aayush Khadka^1,2^, Lucia Pacca^1,2,3^, Jillian Hebert^1,4^, Justin S White^5^, Rita Hamad^6^, Willa D. Brenowitz^3,7^, M. Maria Glymour^8^, Anusha M. Vable^1,2,4^

**Author affiliations**	^1^ Department of Family and Community Medicine, University of California San Francisco

^2^ Philip R. Lee Institute for Health Policy Studies, University of California San Francisco

^3^ Department of Epidemiology and Biostatistics, University of California San Francisco

^4^ School of Public Health, Washington University in St. Louis

^5^ Department of Health Law, Policy and Management, Boston University

^6^ Department of Social and Behavioral Sciences, Harvard T. H. Chan School of Public Health

^7^ Kaiser Permanente Center for Health Research

^8^ Department of Epidemiology, Boston University

**Tables S1-S9**

**Figures S1-S14**

**Table S1 Impact of draft eligibility on self-reported veteran status among men stratified by year of birth**

| **Outcome: Self-reported veteran status** | | | | | | | |
| --- | --- | --- | --- | --- | --- | --- | --- |
| **Year** | **1944** | **1945** | **1946** | **1947** | **1948** | **1949** | **1950** |
| Draft eligible | 3.13 | 2.33 | 5.01 | 9.02 | 5.37 | 10.97 | 14.69 |
|  | [-13.84, 20.10] | [-15.25, 19.91] | [-9.30, 19.31] | [-5.65, 23.68] | [-7.81, 18.56] | [-1.33, 23.26] | [ 3.58, 25.80] |
| N | 150 | 162 | 216 | 198 | 252 | 279 | 305 |
| **Year** | **1951** | **1952** | **1953** | **1954** | **1955** | **1956** |  |
| Draft eligible | 1.36 | 13.30 | 12.12 | 0.39 | -0.10 | -1.48 |  |
|  | [-9.16, 11.87] | [ 2.13, 24.47] | [ 1.41, 22.82] | [-9.09, 9.88] | [-10.20, 9.99] | [-11.48, 8.53] |  |
| N | 276 | 330 | 330 | 311 | 325 | 335 |  |

Notes: Estimates reflect percentage point change in the probability of being a veteran (self-reported) when comparing the draft eligible group with the draft ineligible group. Impact of draft eligibility on veteran status was estimated using a linear regression of self-reported veteran status among men on draft eligibility stratified by the respondent’s year of birth. The regression model included month of birth fixed effects. 95% confidence intervals, shown inside the brackets, were estimated using heteroskedasticity robust standard errors.

**Table S2 Birth cohorts eligible to be drafted by year of the Vietnam draft lottery**

| **Lottery year** | **Eligible birth cohorts** | **Draft threshold number** |
| --- | --- | --- |
| 1969 | 1944-1950 | 195 |
| 1970 | 1951 | 125 |
| 1971 | 1952 | 95 |
| 1972 | 1953 | 95 |
| 1973 | 1954 | 95 |
| 1974 | 1955 | 95 |
| 1975 | 1956 | 95 |

Notes: The Vietnam draft lottery was held once a year between 1969 and 1975. Each lottery had specific birth cohorts of men eligible to be drafted. In each lottery, random numbers between 1 and 366 were assigned to each day-month combination in a year. Then, a threshold number was determined and all men with lottery numbers less than or equal to the threshold were deemed draft eligible. The second column in the table above indicates the birth cohorts eligible for each lottery while the third column shows the threshold number selected by the military for each lottery.

**Table S3 Proportion of item missingness in variables used in the analysis by draft eligibility**

|  | **Draft eligible** | | | |
| --- | --- | --- | --- | --- |
|  | Yes | No | Mean difference | 95% confidence interval |
| Veteran status | - | - | - | - |
| Birth year | - | - | - | - |
| Birth month | - | - | - | - |
| Race and ethnicity | 0.2 | 0.1 | 0.1 | [-0.3, 0.4] |
| Maternal education | 13 | 10.7 | 2.3 | [-3.6, 5.0] |
| Paternal education | 21.4 | 18.4 | 3 | [-3.2, 6.3] |
| Southern US birth | - | - | - | - |
| US birth | - | - | - | - |
| Childhood socioeconomic status | 23.8 | 24.1 | -0.3 | [-3.8, 3.2] |
| Memory score | 0.1 | 0.1 | 0 | [-0.2. 0.3] |

Notes: This table shows the proportion of rows that had missing values for variables that were used in the analysis in some form (either in creating Table 1 or for the main analyses). Mean difference was estimated using a linear regression model with an indicator for missingness as the outcome and the draft eligibility indicator variable as the primary exposure. Confidence intervals were estimated using heteroskedasticity robust standard errors.

**Table S4 Proportion of respondents who died over the study period by draft eligibility status**

|  | **Draft eligible** | | | |
| --- | --- | --- | --- | --- |
|  | Yes | No | Mean difference | 95% confidence interval |
| Primary analytic sample (N = 2,337) |  |  |  |  |
| Died over study period (%) | 13.5 | 13.2 | 0.3 | [-2.5, 3.1] |
| Lost to follow-up over study period (%) | 8.1 | 8.1 | 0 | [-2.2, 2.3] |

Notes: This table shows the proportion of individuals in our primary analytic sample who either died over the study period (1998-2018) or were lost to follow-up for reasons other than death (e.g., dropped from the HRS study). Mean difference was estimated using a linear regression model with an indicator for death or lost to follow-up as the outcome and the draft eligibility indicator variable as the primary exposure. Confidence intervals were estimated using heteroskedasticity robust standard errors.

**Figure S1 Impact of draft eligibility on memory score levels among men with low childhood socioeconomic status**

Notes: This figure illustrates the intention-to-treat effects on memory score levels among men with low childhood socioeconomic status at the mean (“GEE”) and between the 1^st^-99^th^ quantiles (quantiles on the x-axis labeled as “Q10” for the 10^th^ quantile, “Q20” for the 20^th^ quantile, and so forth). The y-axis for both panels represents the change in standardized memory score, i.e., the coefficient from regression models. Effects at the mean were estimated using Generalized Estimating Equations while quantile regression estimates were produced using the Machado and Santos Silva quantile regression estimator. All models controlled for practice effects, year in which the outcome was measured, and month-and-year-of-birth fixed effects.

**Figure S2 Impact of draft eligibility on memory score levels among men with medium childhood socioeconomic status**

Notes: This figure illustrates the intention-to-treat effects on memory score levels among men with medium childhood socioeconomic status at the mean (“GEE”) and between the 1^st^-99^th^ quantiles (quantiles on the x-axis labeled as “Q10” for the 10^th^ quantile, “Q20” for the 20^th^ quantile, and so forth). The y-axis for both panels represents the change in standardized memory score, i.e., the coefficient from regression models. Effects at the mean were estimated using Generalized Estimating Equations while quantile regression estimates were produced using the Machado and Santos Silva quantile regression estimator. All models controlled for practice effects, year in which the outcome was measured, and month-and-year-of-birth fixed effects.

**Figure S3 Impact of draft eligibility on memory score levels among men with high childhood socioeconomic status**

Notes: This figure illustrates the intention-to-treat effects on memory score levels among men with high childhood socioeconomic status at the mean (“GEE”) and between the 1^st^-99^th^ quantiles (quantiles on the x-axis labeled as “Q10” for the 10^th^ quantile, “Q20” for the 20^th^ quantile, and so forth). The y-axis for both panels represents the change in standardized memory score, i.e., the coefficient from regression models. Effects at the mean were estimated using Generalized Estimating Equations while quantile regression estimates were produced using the Machado and Santos Silva quantile regression estimator. All models controlled for practice effects, year in which the outcome was measured, and month-and-year-of-birth fixed effects.

**Figure S4 Impact of draft eligibility on memory score decline rate among men with low childhood socioeconomic status**

Notes: This figure illustrates the intention-to-treat effects on memory score decline among men with low childhood socioeconomic status at the mean (“GEE”) and between the 1^st^-99^th^ quantiles (quantiles on the x-axis labeled as “Q10” for the 10^th^ quantile, “Q20” for the 20^th^ quantile, and so forth). The y-axis for both panels represents the change in standardized memory score, i.e., the coefficient from regression models. Effects at the mean were estimated using Generalized Estimating Equations while quantile regression estimates were produced using the Machado and Santos Silva quantile regression estimator. All models controlled for practice effects, year in which the outcome was measured, and month-and-year-of-birth fixed effects.

**Figure S5 Impact of draft eligibility on memory score decline rate among men with medium childhood socioeconomic status**

Notes: This figure illustrates the intention-to-treat effects on memory score decline among men with medium childhood socioeconomic status at the mean (“GEE”) and between the 1^st^-99^th^ quantiles (quantiles on the x-axis labeled as “Q10” for the 10^th^ quantile, “Q20” for the 20^th^ quantile, and so forth). The y-axis for both panels represents the change in standardized memory score, i.e., the coefficient from regression models. Effects at the mean were estimated using Generalized Estimating Equations while quantile regression estimates were produced using the Machado and Santos Silva quantile regression estimator. All models controlled for practice effects, year in which the outcome was measured, and month-and-year-of-birth fixed effects.

**Figure S6 Impact of draft eligibility on memory score decline rates among men with high childhood socioeconomic status**

Notes: This figure illustrates the intention-to-treat effects on memory score decline among men with high childhood socioeconomic status at the mean (“GEE”) and between the 1^st^-99^th^ quantiles (quantiles on the x-axis labeled as “Q10” for the 10^th^ quantile, “Q20” for the 20^th^ quantile, and so forth). The y-axis for both panels represents the change in standardized memory score, i.e., the coefficient from regression models. Effects at the mean were estimated using Generalized Estimating Equations while quantile regression estimates were produced using the Machado and Santos Silva quantile regression estimator. All models controlled for practice effects, year in which the outcome was measured, and month-and-year-of-birth fixed effects.

**Table S5 Impact of instrumented veteran status on standardized memory score levels and rate of decline by childhood socioeconomic status**

|  | **Point estimate** | **95% confidence intervals** |
| --- | --- | --- |
| **Low childhood socioeconomic status (N = 603)** | | |
| Local Average Treatment Effect |  |  |
| Memory score levels | 0.71 | [-0.78, 2.20] |
| First stage partial F-statistic: 14.01 | | |
| Rate of memory score decline | -0.16 | [-0.42, 0.10] |
| Impact on median memory score |  |  |
| Memory score levels | 0.42 | [-0.13, 0.96] |
| Rate of memory score decline | -0.06 | [-0.23, 0.10] |
| **Total person-wave observations** | 3,576 |  |
| **Medium childhood socioeconomic status (N = 598)** | | |
| Local Average Treatment Effect |  |  |
| Memory score levels | -0.17 | [-1.19, 0.84] |
| First stage partial F-statistic: 23.82 | | |
| Rate of memory score decline | -0.10 | [-0.24, 0.04] |
| Impact on median memory score |  |  |
| Memory score levels | -0.11 | [-0.46, 0.25] |
| Rate of memory score decline | -0.06 | [-0.18, 0.07] |
| **Total person-wave observations** | 3,630 |  |
| **High childhood socioeconomic status (N = 576)** | | |
| Local Average Treatment Effect |  |  |
| Memory score levels | 0.38 | [-0.85, 1.62] |
| First stage partial F-statistic: 5.23 | | |
| Rate of memory score decline | -0.07 | [-0.22, 0.09] |
| Impact on median memory score |  |  |
| Memory score levels | 0.38 | [-0.18, 0.93] |
| Rate of memory score decline | -0.05 | [-0.20, 0.09] |
| **Total person-wave observations** | 3,647 |  |

Notes: Instrumental variables analysis results are presented both for mean memory score (i.e., the Local Average Treatment Effect) and median memory score. The impact at the median is not applicable to just the complier population but to the entire sample.

**Figure S7 Impact of draft eligibility on memory score levels among non-Hispanic White men**

Notes: This figure illustrates the intention-to-treat effects on memory score levels among non-Hispanic White men at the mean (“GEE”) and between the 1^st^-99^th^ quantiles (quantiles on the x-axis labeled as “Q10” for the 10^th^ quantile, “Q20” for the 20^th^ quantile, and so forth). The y-axis for both panels represents the change in standardized memory score, i.e., the coefficient from regression models. Effects at the mean were estimated using Generalized Estimating Equations while quantile regression estimates were produced using the Machado and Santos Silva quantile regression estimator. All models controlled for practice effects, year in which the outcome was measured, and month-and-year-of-birth fixed effects.

**Figure S8 Impact of draft eligibility on memory score levels among non-Hispanic Black men**

Notes: This figure illustrates the intention-to-treat effects on memory score levels among non-Hispanic Black men at the mean (“GEE”) and between the 1^st^-99^th^ quantiles (quantiles on the x-axis labeled as “Q10” for the 10^th^ quantile, “Q20” for the 20^th^ quantile, and so forth). The y-axis for both panels represents the change in standardized memory score, i.e., the coefficient from regression models. Effects at the mean were estimated using Generalized Estimating Equations while quantile regression estimates were produced using the Machado and Santos Silva quantile regression estimator. All models controlled for practice effects, year in which the outcome was measured, and month-and-year-of-birth fixed effects.

**Figure S9 Impact of draft eligibility on memory score levels among Hispanic men**

Notes: This figure illustrates the intention-to-treat effects on memory score levels among Hispanic men at the mean (“GEE”) and between the 1^st^-99^th^ quantiles (quantiles on the x-axis labeled as “Q10” for the 10^th^ quantile, “Q20” for the 20^th^ quantile, and so forth). The y-axis for both panels represents the change in standardized memory score, i.e., the coefficient from regression models. Effects at the mean were estimated using Generalized Estimating Equations while quantile regression estimates were produced using the Machado and Santos Silva quantile regression estimator. All models controlled for practice effects, year in which the outcome was measured, and month-and-year-of-birth fixed effects.

**Figure S10 Impact of draft eligibility on memory score decline rates among non-Hispanic White men**

Notes: This figure illustrates the intention-to-treat effects on memory score decline among non-Hispanic White men at the mean (“GEE”) and between the 1^st^-99^th^ quantiles (quantiles on the x-axis labeled as “Q10” for the 10^th^ quantile, “Q20” for the 20^th^ quantile, and so forth). The y-axis for both panels represents the change in standardized memory score, i.e., the coefficient from regression models. Effects at the mean were estimated using Generalized Estimating Equations while quantile regression estimates were produced using the Machado and Santos Silva quantile regression estimator. All models controlled for practice effects, year in which the outcome was measured, and month-and-year-of-birth fixed effects.

**Figure S11 Impact of draft eligibility on memory score decline rates among non-Hispanic Black men**

Notes: This figure illustrates the intention-to-treat effects on memory score decline among non-Hispanic Black men at the mean (“GEE”) and between the 1^st^-99^th^ quantiles (quantiles on the x-axis labeled as “Q10” for the 10^th^ quantile, “Q20” for the 20^th^ quantile, and so forth). The y-axis for both panels represents the change in standardized memory score, i.e., the coefficient from regression models. Effects at the mean were estimated using Generalized Estimating Equations while quantile regression estimates were produced using the Machado and Santos Silva quantile regression estimator. All models controlled for practice effects, year in which the outcome was measured, and month-and-year-of-birth fixed effects.

**Figure S12 Impact of draft eligibility on memory score decline rate among Hispanic men**

Notes: This figure illustrates the intention-to-treat effects on memory score decline among Hispanic men at the mean (“GEE”) and between the 1^st^-99^th^ quantiles (quantiles on the x-axis labeled as “Q10” for the 10^th^ quantile, “Q20” for the 20^th^ quantile, and so forth). The y-axis for both panels represents the change in standardized memory score, i.e., the coefficient from regression models. Effects at the mean were estimated using Generalized Estimating Equations while quantile regression estimates were produced using the Machado and Santos Silva quantile regression estimator. All models controlled for practice effects, year in which the outcome was measured, and month-and-year-of-birth fixed effects.

**Table S6 Impact of instrumented veteran status on standardized memory score levels and rate of decline by race and ethnicity**

|  | **Point estimate** | **95% confidence intervals** |
| --- | --- | --- |
| **Non-Hispanic White men (N = 1,369)** | | |
| **Impact of instrumented veteran status on memory score outcomes** | | |
| Local Average Treatment Effect |  |  |
| Memory score levels | -0.002 | [-0.24, 0.23] |
| First stage partial F-statistic: 7.46 | | |
| Rate of memory score decline | -0.11 | [-0.20, 0.03] |
| Impact on median memory score |  |  |
| Memory score levels | 0.08 | [-0.09, 0.25] |
| Rate of memory score decline | -0.07 | [-0.13, -0.01] |
| **Total person-wave observations** | 8,172 |  |
| **Non-Hispanic Black men (N = 491)** | | |
| **Impact of instrumented veteran status on memory score outcomes** | | |
| Local Average Treatment Effect |  |  |
| Memory score levels | -2.10 | [-18.06, 13.85] |
| First stage partial F-statistic: 1782.36 | | |
| Rate of memory score decline | -1.43 | [-19.75, 16.90] |
| Impact on median memory score |  |  |
| Memory score levels | -1.34 | [-410.80, 408.13] |
| Rate of memory score decline | -0.25 | [-1.20, 0.70] |
| **Total person-wave observations** | 2,422 |  |
| **Hispanic men (N = 389)** | | |
| **Impact of instrumented veteran status on memory score outcomes** | | |
| Local Average Treatment Effect |  |  |
| Memory score levels | -2.93 | [-28.53, 22.67] |
| First stage partial F-statistic: 0.053 | | |
| Rate of memory score decline | -0.49 | [-2.71, 1.74] |
| Impact on median memory score |  |  |
| Memory score levels | 1.50 | [-97.74, 100.73] |
| Rate of memory score decline | -0.06 | [-20.61, 20.50] |
| **Total person-wave observations** | 1,992 |  |

Notes: Instrumental variables analysis results are presented both for mean memory score (i.e., the Local Average Treatment Effect) and median memory score. The impact at the median is not applicable to just the complier population but to the entire sample.

**Table S7 Impact of instrumented veteran status on standardized memory score levels and rate of decline among all men born between 1944 and 1953**

|  | **Point estimate** | **95% confidence intervals** |
| --- | --- | --- |
| Local Average Treatment Effect |  |  |
| Memory score levels | 0.37 | [-0.30, 1.04] |
| First stage partial F-statistic: 15.77 | | |
| Rate of memory score decline | -0.10 | [-0.21, 0.02] |
| Impact on median memory score |  |  |
| Memory score levels | 0.22 | [0.01, 0.44] |
| Rate of memory score decline | -0.08 | [-0.14, -0.01] |
| **Total person-wave observations** | 18,058 |  |

Notes: Instrumental variables analysis results are presented both for mean memory score (i.e., the Local Average Treatment Effect) and median memory score. The impact at the median is not applicable to just the complier population but to the entire sample.

**Figure S13 Impact of draft eligibility on memory score among all men born between 1944 and 1953**

Notes: Both panels illustrate the intention-to-treat effects for men born between 1944 and 1953 at the mean (“GEE”) and between the 1^st^-99^th^ quantiles (quantiles on the x-axis labeled as “Q10” for the 10^th^ quantile, “Q20” for the 20^th^ quantile, and so forth). The y-axis for both panels represents the change in standardized memory score, i.e., the coefficient from regression models. Panel A shows draft eligibility’s effect on memory score levels and panel B shows draft eligibility’s effect on the rate of memory score decline. Effects at the mean were estimated using Generalized Estimating Equations while quantile regression estimates were produced using the Machado and Santos Silva quantile regression estimator. All models controlled for practice effects, year in which the outcome was measured, and month-and-year-of-birth fixed effects.

**Table S8 Impact of instrumented veteran status restricted to Vietnam lottery years on standardized memory score levels and rate of decline**

|  | Point estimate | 95% confidence interval |
| --- | --- | --- |
| **Local Average Treatment Effect** |  |  |
| Memory score levels | 0.12 | [-0.57. 0.80] |
| First stage partial F-statistic: | 24.27 |  |
| Rate of memory score decline | -0.27 | [-0.52, -0.02] |
| **Impact on median memory score** |  |  |
| Memory score levels | 0.13 | [-0.09, 0.34] |
| Rate of memory score decline | -0.18 | [-0.37, 00] |
| Total person-wave observations | 13,031 |  |

Notes: Instrumental variables analysis results are presented both for mean memory score (i.e., the Local Average Treatment Effect) and median memory score. The impact at the median is not applicable to just the complier population but to the entire sample.

**Table S9 Impact of instrumented veteran status on total word recall score**

|  | **Point estimate** | **95% confidence intervals** |
| --- | --- | --- |
| Local Average Treatment Effect |  |  |
| Total word recall score levels | 0.15 | [-2.01, 2.30] |
| First stage partial F-statistic: 19.42 | | |
| Rate of total word recall score decline | -0.39 | [-0.72, -0.05] |
| Impact on median total word recall score |  |  |
| Total word recall score levels | 0.17 | [-1.09, 1.43] |
| Rate of total word recall score decline | -0.27 | [-0.56, 0.03] |
| **Total person-wave observations** | 11,751 |  |

Notes: Instrumental variables analysis results are presented both for mean total word recall score (i.e., the Local Average Treatment Effect) and median total word recall score. The impact at the median is not applicable to just the complier population but to the entire sample.

**Figure S14 Impact of draft eligibility on total word recall score among men born between 1947 and 1953**

Notes: Both panels illustrate the intention-to-treat effects at the mean (“GEE”) and between the 1^st^-99^th^ quantiles (quantiles on the x-axis labeled as “Q10” for the 10^th^ quantile, “Q20” for the 20^th^ quantile, and so forth). The y-axis for both panels represents the change in total word recall score, i.e., the coefficient from regression models. Panel A shows draft eligibility’s effect on memory score levels and panel B shows draft eligibility’s effect on the rate of memory score decline. Effects at the mean were estimated using Generalized Estimating Equations while quantile regression estimates were produced using the Machado and Santos Silva quantile regression estimator. All models controlled for practice effects, year in which the outcome was measured, and month-and-year-of-birth fixed effects.
